# Invertebrate and vertebrate predation rates in a hyperarid ecosystem following an oil spill

**DOI:** 10.1002/ece3.7978

**Published:** 2021-08-13

**Authors:** Marco Ferrante, Daniella Möller, Gabriella Möller, Esteban Menares, Yael Lubin, Michal Segoli

**Affiliations:** ^1^ Mitrani Department of Desert Ecology Blaustein Institutes for Desert Research Ben‐Gurion University of the Negev Midreshet Ben‐Gurion Israel; ^2^ Ce3C ‐ Centre for Ecology, Evolution and Environmental Changes Azorean Biodiversity Group Faculty of Agricultural Sciences and Environment University of the Azores Angra do Heroísmo Portugal; ^3^ Department of Ecology Brandenburg University of Technology Cottbus‐Senftenberg Cottbus Germany

**Keywords:** acacia, biotic interactions, ecological functions, Evrona Nature Reserve, oil pollution, sentinel prey

## Abstract

Extreme temperatures and scarce precipitation in deserts have led to abiotic factors often being regarded as more important than biotic ones in shaping desert communities. The presumed low biological activity of deserts is also one reason why deserts are often overlooked by conservation programs. We provide the first quantification of predation intensity from a desert ecosystem using artificial sentinel prey emulating caterpillars, a standardized monitoring tool to quantify relative predation pressure by many invertebrate and vertebrate predators. The study was conducted in a protected natural area affected by oil spills in 1975 and 2014; hence, we assessed the potential effects of oil pollution on predation rates. We found that predation was mostly due to invertebrate rather than vertebrate predators, fluctuated throughout the year, was higher at the ground level than in the tree canopy, and was not negatively affected by the oil spills. The mean predation rate per day (12.9%) was within the range found in other ecosystems, suggesting that biotic interactions in deserts ought not to be neglected and that ecologists should adopt standardized tools to track ecological functions and allow for comparisons among ecosystems.

## Graphical abstract

Extreme environments such as deserts are expected to have low biodiversity and, therefore, reduced biotic interactions. We addressed this assumption by measuring invertebrate and vertebrate predation rates in a hyper‐arid desert using a standardized monitoring tool that allows recording predator activity in a quantitative, comparable way. We found that the mean predation rate (12.9% per day) is within the range found in other ecosystems, suggesting that this biotic function is no less important in deserts than elsewhere.

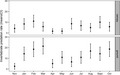

## 1 INTRODUCTION

Surviving in desert environments can be challenging. Deserts are characterized by a deficit of available water, and other abiotic factors such as high temperature and low nutrient concentrations can also be limiting (Laity, 2009; Ward, 2016). Moreover, the distribution of resources in deserts is highly patchy in space and irregular in time (Megías et al., 2011). Desert flora and fauna often respond to these harsh conditions with spectacular physiological and behavioral adaptations (Slobodkin, 1989). Because the extreme abiotic factors and their effects on desert‐dwelling organisms are evident, biotic interactions have often been assumed to be less important in explaining how desert ecosystems function and have thus received less attention (Megías et al., 2011).

Available research, however, has shown that biotic interactions can have strong influences on structuring desert communities and ecosystem functioning (Ward, 2016). In fact, notwithstanding their characteristic low productivity, deserts can host surprisingly high biodiversity and numbers of trophic links (Durant et al., 2012; Polis, 1991a; Ward, 2016). Predators, in particular, are suggested to play a major role in structuring desert ecosystems through direct and indirect effects (Abramsky et al., 1992; Ayal, 2007; Henschel, 1990; Loria et al., 2008; Polis et al., 1998; Segoli et al., 2016). Nevertheless, while predator diversity, abundance, and biomass suggest that predation intensity in deserts may be important (Polis, 1991b; Rundel & Gibson, 2005; Shachak et al., 2005), quantitative comparable estimates for predation rates are still lacking (Lövei & Ferrante, 2017).

Due to the limiting conditions, deserts are especially vulnerable to anthropogenic disturbance (Lovich & Bainbridge, 1999) and may take long to recover (Guo, 2004). Human activities that affect the biological soil crust (Belnap & Lange, 2013), natural vegetation (Nothers et al., 2017; Seifan, 2009), and water availability (Ward & Rohner, 1997) are particularly harmful, as they can disrupt biological processes and make already limited resources inaccessible. For example, oil spills are a major cause of environmental pollution (Rahman et al., 2002), with dramatic effects on flora and fauna (Baker, 1970; Odukoya et al., 2019; Stadler & Buteler, 2009). While these effects have been studied extensively in marine environments, very little is known about the effect of oil pollution on desert organisms. Consequently, despite deserts being the terrestrial ecosystem with the major number of oil spills (Nicolotti & Egli, 1998), they are usually underrepresented in conservation efforts (Durant et al., 2014; Schimel, 2010).

To fill the abovementioned gaps, we investigated the effects of oil pollution in the Evrona Nature Reserve, a hyperarid desert ecosystem located in the Arava Rift Valley of Israel. In this area, two large oil spills occurred in 1975 and 2014, during which around 10,000 m^3^ and 5,000 m^3^ of crude oil, respectively, leaked into the area jeopardizing the entire ecosystem (Golan et al., 2016). Oil pollution has been shown to affect the metabolism, recruitment, and germination of keystone tree species in this area (Ferrante et al., 2021; Golan et al., 2016; Nothers et al., 2017) and may negatively affect insect biodiversity (Möller et al., 2020) with potential cascading effects on biotic interactions. We focused our study on predation, a fundamental ecosystem function (Hairston et al., 1960). However, quantifying predation is challenging, especially on arthropod prey, as no trace of the predatory event is usually left.

Sentinel prey can be used to obtain quantitative data on predation intensity on invertebrates (Howe et al., 2009; Lövei & Ferrante, 2017; Meyer et al., 2015). This method consists of placing a known number of real or artificial prey items (*i.e*., the sentinels) in a habitat and monitoring how many have been attacked or disappeared after a given exposure time to assess the activity of diurnal and nocturnal predators. As the artificial set‐up of the sentinel prey is different in many aspects from natural prey (*e.g*., prey densities, prey behavior), this method does not provide absolute predation rates, but it is suitable for standardized, relative comparisons (see Lövei & Ferrante, 2017 for a review of the method). Artificial “caterpillars” made of plasticine have been used for this purpose in both temperate and tropical ecosystems. As artificial caterpillars only vaguely resemble real ones, animals whose predator behavior is triggered by prey movements or chemical cues are likely overlooked by this method. Yet, a wide range of vertebrates and invertebrates that use visual cues to seek their prey typically attack artificial caterpillars and can leave diagnostic marks on the plasticine that allow identification (Low et al., 2014).

We used this method to quantify invertebrate and vertebrate predation rates monthly throughout one year on oil‐polluted and unpolluted *Vachellia* trees, keystone species in this habitat, both at ground and tree canopy levels. Our aims were two‐fold: (a) to provide the first quantitative measures of variation in predation intensity on arthropods in a desert ecosystem and (b) to assess the effects of large‐scale terrestrial oil spills on predation rates. We predicted that the oil spills would negatively affect ecological functioning, reducing predation intensity in polluted plots.

## 2 METHODS

### 2.1 Study site

**FIGURE 1:**
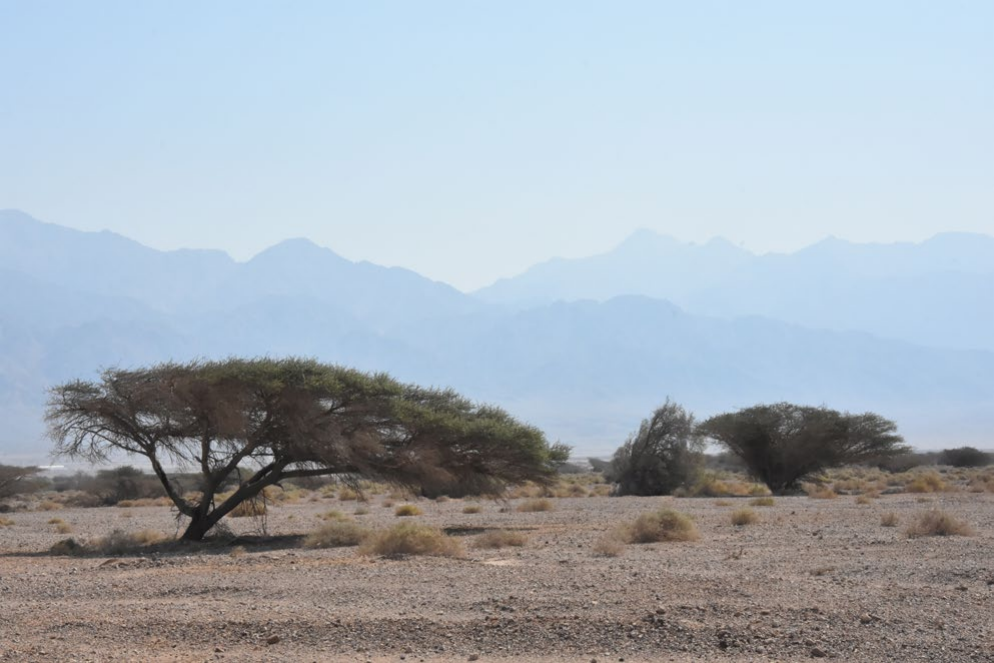
*Vachellia* (formerly *Acacia*) trees in Evrona Nature Reserve, southern Israel

The study site was located in the Evrona Nature Reserve, a large (16.723 km^2^) protected area in southern Israel (29°40′N, 35°00′E; Figure 1). This area is a hyperarid desert ecosystem characterized by 25–50 mm annual precipitation and average summer temperatures around 31°C (Bruins et al., 2012). The reserve was affected by two major oil spills, in 1975 and 2014 due to leaks in the Eilat‐Ashkelon pipeline. The polluted soil is still visible even in the site affected by the 1975 oil spill, as no remediation measures were undertaken. In the site affected by the 2014 oil spill, the polluted soil was tilled in December 2014 and 2019, in an attempt to improve its hydrological properties. Due to the irregularity of the soil surface, the oil spread unevenly in the reserve and was naturally canalized into the normally dry watercourses (“wadis”), leaving some other areas free of pollution. The two tree species present in the area are *Vachellia* (formerly *Acacia*) *tortilis* (Forssk.) and *V. raddiana* (Savi), which provide shade, refuge, and food for the local fauna, and are considered keystone species (Munzbergova & Ward, 2002; Stavi et al., 2014). *Vachellia raddiana* is considered a subspecies of *V. tortilis*, but in Israel, it is regarded as a separate species because of its distinctive genetic, morphological, and ecological characteristics (Ferrante et al., 2020; Rodger et al., 2018; Zohary, 1972). The populations of both tree species are in decline due to both direct and indirect anthropogenic causes (Shrestha et al., 2002; Ward & Rohner, 1997).

### 2.2 Study design

While *V. tortilis* is widely distributed within the reserve, *V. raddiana* is scarce in the area reached by the 1975 oil spill. Hence, we selected 30 trees (458.7 ± 189.9 cm height, mean ± *SD*): 10 *V. tortilis* trees in the area impacted by the 1975 oil spill, and 10 *V. tortilis* and 10 *V*.* raddiana* trees in the area impacted by the 2014 oil spill. Half of the selected trees were located directly within the flow path of the oil (henceforth "polluted" trees). The remaining trees were located outside the path taken by the oil (henceforth "unpolluted" trees). *Vachellia* trees have both superficial roots and deep taproots (Sher et al., 2010; Winters et al., 2015, 2018). Deep taproots can extend well below the oil penetration depth (about 0.3 m; Gordon et al., 2018), but the superficial roots likely would be affected by the oil spill. The areas affected by the 1975 and 2014 oil spill were approximately 5 km distant from each other.

### 2.3 Measuring predation

**FIGURE 2:**
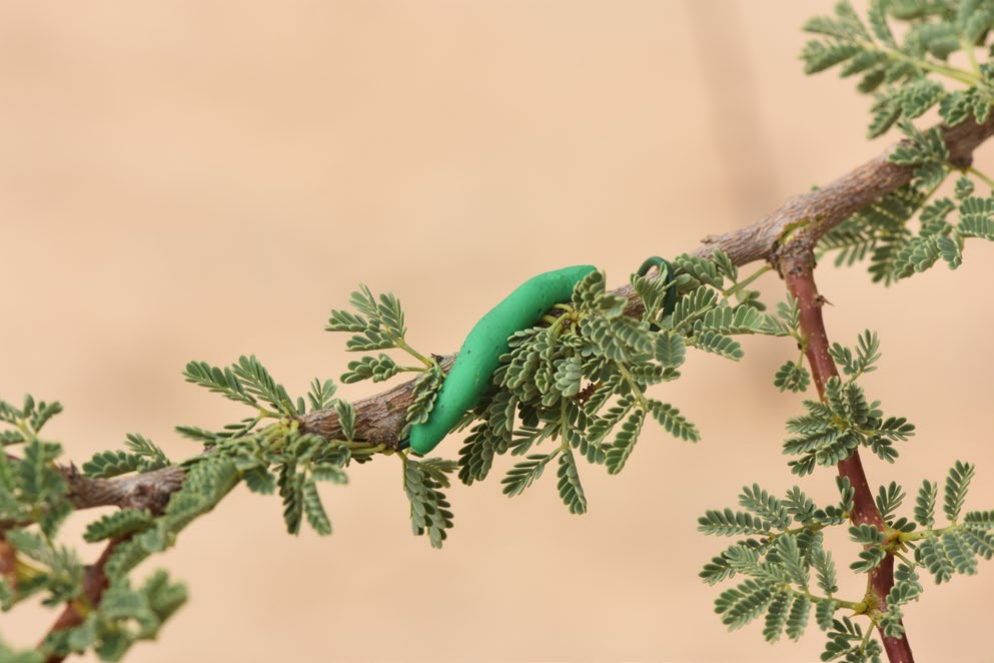
Artificial caterpillar exposed on the branch of a *Vachellia* tree

Between November 2018 and October 2019 (except for December 2018), we quantified predation intensity monthly, at ground and tree canopy level, for each of the selected trees. We modeled artificial “caterpillars” using green plasticine (Smeedi plus, V. nr. 776,609, Denmark), 20 mm long, 3 mm diameter, and prepared in the laboratory following Howe et al. (2009). For each sampling event and on each tree, we placed four caterpillars at ground level near the tree trunk, and four caterpillars in the canopy on four different branches at 1.5–2 m above ground, 50–70 cm from each other, and always in the shade to avoid direct sunlight (Figure 2). In this way, it was possible to quantify the activity of both ground‐active (*e.g.,* ground beetles, ants, lizards, and mammals) and canopy‐active predators (*e.g.,* birds, clerid beetles), which may represent different species belonging to separate communities (Stork & Grimbacher, 2006). Sentinel prey at ground level were fixed to a piece of reed using super glue and placed on the ground, while sentinel prey on trees were directly glued to the upper side of selected small branches. After 24 hr, all caterpillars were inspected for predation marks, which were all identified by a person with extensive experience with the method (MF). If a caterpillar was attacked both by a vertebrate and an invertebrate, a predation event was counted in both predator categories. For each of the eleven sampling occasions, we used 240 caterpillars (*i.e*., 8 caterpillars/tree x 30 trees), for a total of 2,640 sentinel prey during the year, of which 30 (1.14%) were lost and were therefore excluded from the analysis.

### 2.4 Statistical analysis

We analyzed overall predation rates, and invertebrate and vertebrate predation rates separately, using three generalized linear mixed models with binomial distribution and logit link function. The starting models included month (from Nov 2018 to Oct 2019), site (the areas affected by the 1975 and 2014 oil spills, respectively), tree status (polluted vs. unpolluted), and prey position (ground level vs. canopy) as fixed factors, and tree ID as a random factor to take into account repeated measures from the same tree. To test if there was a difference in overall predation rates between the two tree species, we specified an additional model with only tree species as fixed factor and tree ID as a random factor. We then analyzed the tree species separately, as only *V. tortilis* was sampled in the 1975 oil spill site, while both species were sampled in the 2014 site, and thus, the design was not fully factorial. Model selection was done by comparing the Akaike Information Criterion values (Akaike, 1998), and random factors were always kept in all models (Table S1). Tukey's post hoc test was used to compare a categorical factor with more than two levels (*i.e*., month) using the R package *lsmeans* (Lenth, 2016). All analyses were performed using the statistical software R (R Core Team, 2019).

## 3 RESULTS

### 3.1 Overall predation

Of the artificial caterpillars exposed, 12.9% (336/2610) showed attack marks after 24 hr. Most of the attacks were by arthropods (72.6% of the marked caterpillars), followed by birds (19.6%), and small mammals (6.3%). In four cases (1.2%), we could not identify the type of predator. Four caterpillars showed marks by both arthropods and birds. The overall (invertebrate and vertebrate predatory marks together) mean predation rate per tree per day over the year was 12.87% (*SD* = 4.55%, *n* = 30). The model where only tree species as fixed factor and tree ID as a random factor indicated that predation rates on *V*. *tortilis* (mean % per day ±*SD* % per day; 12.45 ± 4.33%, *n* = 20) and *V*. *raddiana* (13.71 ± 5.10%, *n* = 10) were not significantly different (GLMM, *z* = −0.71, *p* = 0.48). The best model for overall predation included month and prey position as fixed factors and tree ID as a random factor (Table S2). Overall predation rate at ground level (16.84 ± 7.85%, *n* = 30) was almost twice the overall predation rate in the tree canopy (8.90 ± 5.01%, *n* = 30, GLMM, *z* = 6.060, *p* < 0.001). For the temporal analysis, predation rates were significantly lower in April than in February (Tukey's post hoc, *z* = 3.737, *p* = 0.01) and September (Tukey's post hoc, *z* = −3.407, *p* = 0.04), with the last two being the highest monthly rates measured. There were no significant differences in overall predation rates between any of the other months. No significant differences were detected between the areas affected by the 1975 and 2014 oil spills (GLMM, *z* = 0.144, *p* = 0.89).

### 3.2 Invertebrate predation rates

**FIGURE 3:**
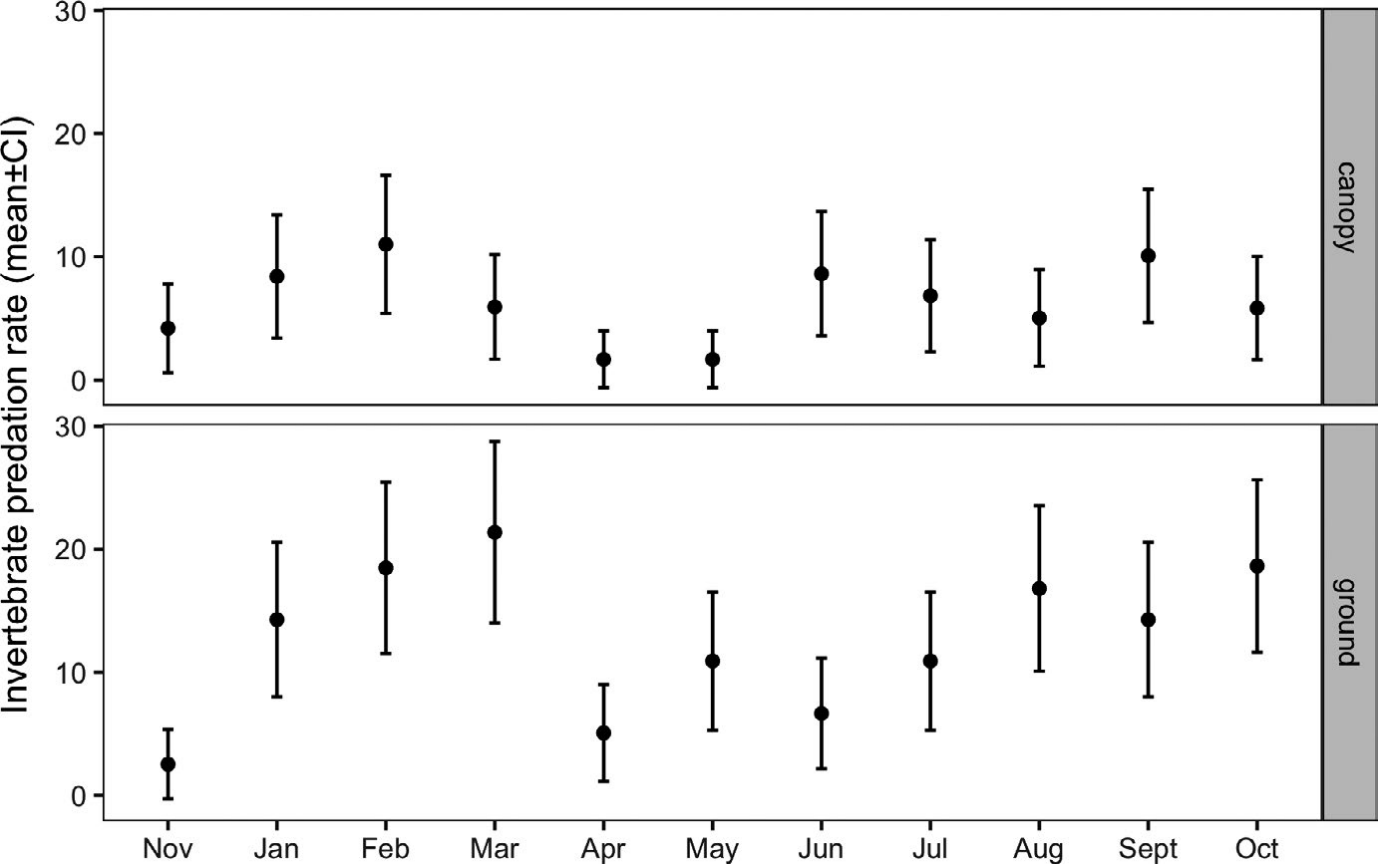
Observed invertebrate predation rates (mean ± 95% CI, *n* = 120 caterpillars per position during each sampling event) on *Vachellia* trees at ground and canopy levels in Evrona Nature Reserve, southern Israel, between November 2018 and October 2019

Invertebrate predation rate was almost three times higher (9.50 ± 3.50%, *n* = 30) than vertebrate predation rate (3.37 ± 2.44%, *n* = 30). The best model for invertebrate predation included only month and prey position as fixed factors and tree ID as a random factor (Table S3). Predation rates were significantly higher at ground level (12.70 ± 5.51%, *n* = 30) than in the tree canopy (6.29 ± 4.36%, *n* = 30; GLMM, *z* = 5.56, *p* < 0.001). Predation rates in April were significantly lower than in early spring (Feb‐Mar) and early autumn (Sept‐Oct) (Figure 3, Table S4). No significant differences were detected between the site affected by the 1975 oil spill (9.26 ± 3.01%, *n* = 10) and the site affected by the 2014 oil spill (9.62 ± 3.80%, *n* = 20; GLMM, *z* = 0.273, *p* = 0.785), nor between polluted (9.07 ± 3.45%, *n* = 15) and unpolluted trees (9.93 ± 3.63%, *n* = 15; GLMM, *z* = −0.694, *p* = 0.488).

### 3.3 Vertebrate predation rates

**FIGURE 4:**
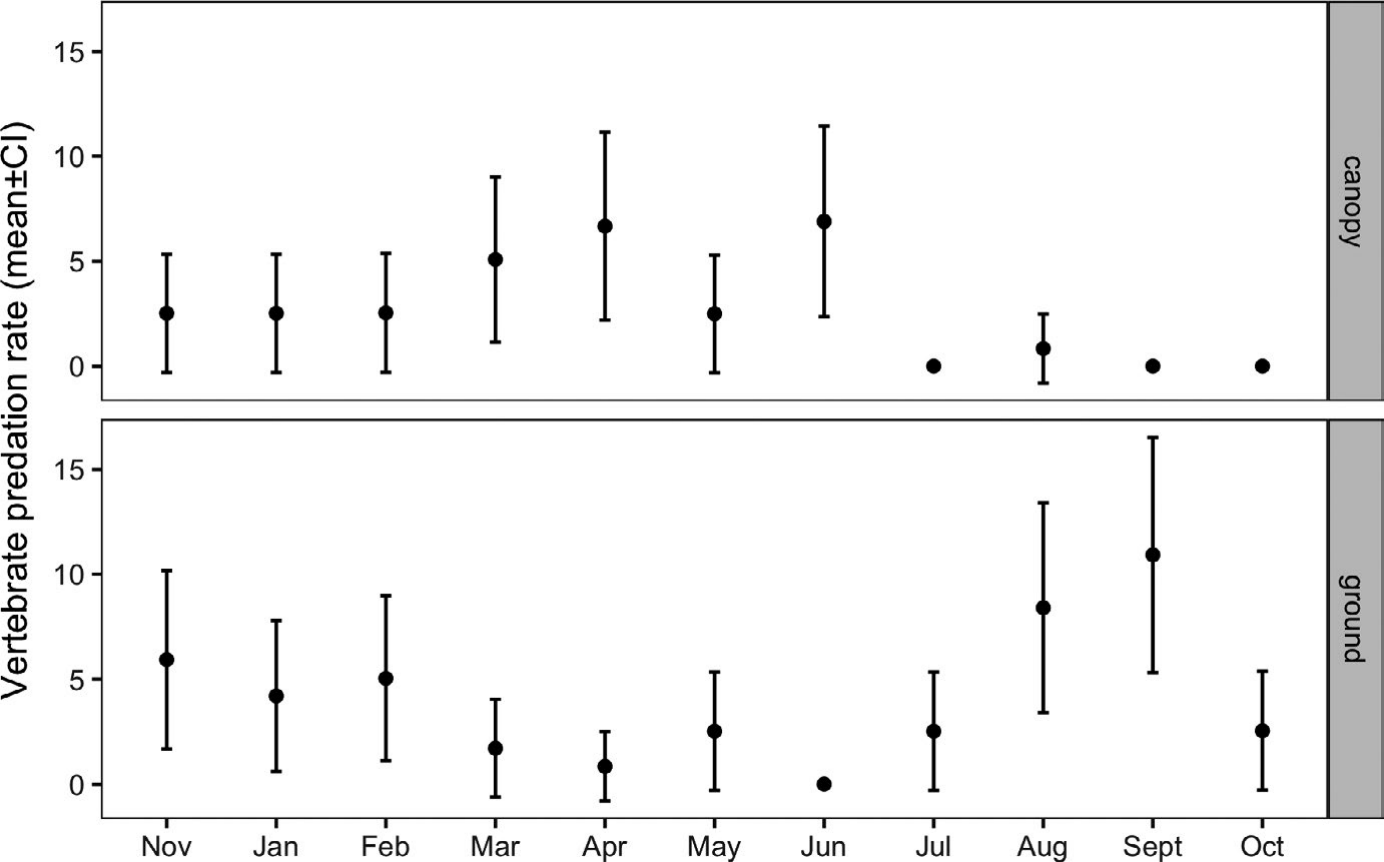
Observed vertebrate predation rates (mean ± 95% CI, *n* = 120 caterpillars per position during each sampling event) on *Vachellia* trees at ground and canopy levels in Evrona Nature Reserve, southern Israel, between November 2018 and October 2019

The best model for vertebrate predation included only prey position (ground vs. canopy) and tree status (oil‐polluted vs. unpolluted) as fixed factors and tree ID as a random factor (Table S5). Similar to invertebrate predation, vertebrate predation rate was significantly higher at ground level (4.06 ± 4.46%, *n* = 30) than in the canopy (2.68 ± 2.43%, *n* = 30; GLMM, *z* = 1.96, *p* = 0.05). Most of the vertebrate predation was by birds (2.37 ± 3.63%, *n* = 30, at ground level, and all cases in the canopy). Unexpectedly, vertebrate predation rates were significantly higher on polluted trees (4.22 ± 2.59%, *n* = 30) than on unpolluted trees (2.52 ± 2.02%, *n* = 30; GLMM, *z* = 2.07, *p* = 0.038). Predation rates were not statistically different between months, despite apparent seasonal trends. Predation rates were nearly constant between Nov‐Feb. Between Mar–Jul, predation rates in the canopy increased but decreased on the ground. Between Jul–Oct, this pattern was reversed: Predation rates in the canopy declined, while predation rate on the ground peaked (Figure 4).

## 4 DISCUSSION

This study provides the first quantification of invertebrate and vertebrate predation rates in a desert ecosystem as a response to an ecological disaster, using a standardized method (artificial sentinel prey). It is also the first assessment with this method of year‐round variability in predation intensity on arthropods in a natural ecosystem.

Contrary to our hypothesis, we found no evidence for a negative effect of the oil spills on predation rates. These results are similar to those observed in the same area for predation rates on *Vachellia* seeds, another ecological function that seemed to be unaffected by the oil spill (Ferrante et al., 2020). One possible explanation for these results is that the ecosystem had already recovered four years after the last (2014) oil spill. Indeed, the arthropod communities of salt marshes affected by oil pollution seemingly recovered in one year after an oil spill (McCall & Pennings, 2012). In the Evrona Nature Reserve, healthy, unpolluted trees may have acted as refuge for animals during the oil spill, allowing a fast recolonization of the previously polluted vegetation. We cannot say, however, whether any species disappeared and were permanently lost at the time the oil spill occurred, as comprehensive background data for the arthropod communities in this area are lacking. Unexpectedly, we found that vertebrate predation was higher on the oil‐polluted than on the unpolluted trees. We find it more likely that higher water availability to the polluted trees due to their central location in the streambed, rather than direct effects of the oil pollution, accounted for this pattern. Although our hypothesis that oil pollution would reduce the activity both invertebrate and vertebrate predators was not confirmed, oil pollution is a major threat to this ecosystem: It was shown to change the soil microbial community (Girsowicz et al., 2018) and parasitoid abundance (Möller et al., 2020), as well as to reduce *Vachellia* seed germination (Tran et al., 2018), seedling recruitment (Nothers et al., 2017), and to modify the tree metabolism (Ferrante et al., 2021).

We found that predation by invertebrates was more frequent than predation by vertebrates. In deserts, ground‐active invertebrates are one of the most diverse guilds (Ward, 2016) and one of the most important in terms of biomass (Rundel & Gibson, 2005), which can explain the high invertebrate predation rates found in Evrona Nature Reserve, especially at the ground level. Invertebrate predation in the canopy may have been due mostly to solifuges (Arachnida, Solifuga), which often hunt on the tree trunk, checkered beetles (Cleridae, *Eucymatodera* cf., *Opilo* cf.), and tree‐active carabids such as *Calodromius henoni*, which were observed in this area throughout the year (Salman et al., 2020). Solifuges and many desert beetles such as carabids and tenebrionids readily attacked artificial prey under laboratory conditions, leaving similar marks to those observed in the field (M Ferrante personal observation). Birds were the main vertebrate predators in this study, while predatory marks by mammals were rare, possibly because most desert rodents, such as gerbils, are mainly seed predators (Ward, 2016). These results are in accordance with previous studies that suggested that birds are important predators in desert ecosystems and may have a large impact on the prey community (Shachak et al., 2005). For example, in the Coachella Valley desert in the USA, 49 of 54 resident bird species were observed feeding on insects (Polis, 1991a).

Additionally, we found that predation rates fluctuated significantly during the year and were higher at ground level than in the tree canopy. The complex seasonal fluctuations in both invertebrate and vertebrate predation rates are perhaps expected in ecosystems such as deserts, where resources are strongly limited in time and space (Megías et al., 2011). Invertebrate predation both in the canopy and on the ground peaked during early spring, decreased in late spring, and then gradually increased until early autumn. This might reflect changes in the abundance and activity of the main predatory species, although these were not quantified in this study. Indeed, desert habitats are characterized by a high seasonal species turnover (Forbes et al., 2005).

Notwithstanding the extreme abiotic characteristics of this environment, we found that the overall mean standardized predation rate on a *Vachellia* tree in this desert ecosystem was 12.9% per day. This was lower than the predation rates found using the same methodology in temperate forests (Ferrante et al., 2017) and cultivated habitats (Ferrante et al., 2017; González et al., 2020; Mansion‐Vaquié et al., 2017; Meyer et al., 2019), and slightly higher than the average predation rate in maize crops in Europe (Ferrante et al., 2019). However, these differences are likely to be smaller, considering that we calculated predation rate over an entire year, while the predation rates obtained for other ecosystems are instead “snapshots” of predation intensity, usually restricted to the peak season of predator activity (*i.e*., spring and summer). In fact, when looking at predation rates at the peak season in our system (23%–24% in spring and 20%–25% in autumn) we find values that are well within the range observed in other ecosystems (Lövei & Ferrante, 2017). Moreover, the overall daily predation rate recorded in the tree canopy of *Vachellia* trees (8.9%) was higher than the predation rates registered in temperate regions (Aslam et al., 2020; Barbaro et al., 2012; Bereczki et al., 2015; Gunnarsson et al., 2018; Low et al., 2014a; Zverev et al., 2020), although lower than in most tropical forests (Denan et al., 2020; Liu et al., 2020; Lövei & Ferrante, 2017). This may be explained by *Vachellia* being the only trees present in this ecosystem and therefore an important habitat for arthropods (Nothers et al., 2017), leading to high predation rates relative to some of the plants sampled in other ecosystems.

This study demonstrated that plasticine sentinel prey can be used effectively to obtain predation rates also in arid environments, despite the extreme temperatures. However, our estimates of predation intensity are likely to be conservative. This is because several agents of mortality for caterpillars are probably underestimated by this method. For example, parasitoid wasps, which are diverse in this ecosystem (Möller et al., 2020), mostly rely on chemical cues (Godfray, 1994) and are unlikely to attack artificial prey. Also, spiders, which are important predators in deserts, mostly attack moving prey (Persons & Uetz, 1997). Given the dire status of biodiversity at global scale (Tittensor et al., 2014), ecologists ought to adopt and further develop new monitoring tools to quantify ecological functions and allow direct comparisons between ecosystems. Providing quantitative data about the intensity of those functions in combination with traditional structural biodiversity assessments is essential for our understanding of ecosystems and to track their health. This is particularly important for ecosystems with extreme climates, as they are expected to be greatly affected by climate change and anthropogenic disturbance (Sala et al., 2000).

## CONFLICT OF INTEREST

The authors disclose no conflicts of interest.

## AUTHOR CONTRIBUTIONS

**Marco Ferrante:** Conceptualization (lead); Formal analysis (lead); Investigation (lead); Writing‐original draft (lead); Writing‐review & editing (lead). **Daniella Möller:** Formal analysis (supporting); Investigation (equal); Writing‐review & editing (supporting). **Gabriella Möller:** Investigation (equal); Writing‐review & editing (supporting). **Esteban Menares:** Investigation (equal); Writing‐review & editing (supporting). **Yael Lubin:** Conceptualization (equal); Resources (lead); Supervision (lead); Writing‐original draft (supporting); Writing‐review & editing (supporting). **Michal Segoli:** Conceptualization (equal); Resources (lead); Supervision (lead); Writing‐original draft (supporting); Writing‐review & editing (supporting).

## Supporting information

Table S1‐S5

## Data Availability

The dataset used for this analysis is archived in Dryad https://doi.org/10.5061/dryad.s1rn8pk86.
