# Supplementary material for: Invertebrate and vertebrate predation rates in a hyperarid ecosystem following an oil spill
Source: Ecol Evol. 2021 Aug 13;11(17):12153–60. doi: 10.1002/ece3.7978 (PMC8427564; doi:10.1002/ece3.7978)
Supplement: Supplementary file 1 — Table S1‐S5 [file ECE3-11-12153-s001.doc]

Supplementary information for the article **Invertebrate and vertebrate predation rates in a hyper-arid ecosystem following an oil spill** by Ferrante Marco, Möller Daniella, Möller Gabriella, Menares Esteban, Lubin Yael, Segoli Michal.

|  | Table S1. Backward model selection based on AIC starting with a full model with no interactions. Best models have the lowest AIC. | | | |  |
| --- | --- | --- | --- | --- | --- |
|  | **Model response** | **Fixed factors** | **Random effect** | **AIC** |  |
|  | Overall predation | Month + Site + Treatment + Position | TreeID | 1956.246 |  |
|  |  | Month + Treatment + Position | TreeID | 1954.266 |  |
|  |  | Month + Position | TreeID | 1952.368 |  |
|  | Arthropod predation | Month + Site + Treatment + Position | TreeID | 1588.009 |  |
|  |  | Month + Site + Position | TreeID | 1586.469 |  |
|  |  | Month + Position + Treatment | TreeID | 1586.071 |  |
|  |  | Month + Position | TreeID | 1584.544 |  |
|  | Vertebrate predation | Month + Site + Treatment + Position | TreeID | 774.6388 |  |
|  |  | Month + Position + Treatment | TreeID | 772.6495 |  |
|  |  | Position + Treatment | TreeID | 766.2609 |  |
| Table S2. Output of the best generalized linear mixed model explaining overall predation rates on *Vachellia* trees in Evrona Nature Reserve, southern Israel, during 2018-2019. | | | | | |

|  | **Effect** | | | | | | |  |  | |
| --- | --- | --- | --- | --- | --- | --- | --- | --- | --- | --- |
|  | **Fixed:** | | | |  | **Random  (tree ID, n = 30):** | | | |  |
|  | **Estimate** | **SE** | **z-value** | **p-value** |  | | **Variance** | **St. Dev** | | |
| (Intercept) | -2.839 | 0.253 | -11.21 | < 0.001 |  | | 0.068 | 0.261 | | |
| January | 0.636 | 0.299 | 2.128 | 0.033 |  | |  |  | | |
| February | 0.950 | 0.289 | 3.289 | 0.001 |  | |  |  | | |
| March | 0.815 | 0.293 | 2.776 | 0.006 |  | |  |  | | |
| April | -0.184 | 0.345 | -0.534 | 0.593 |  | |  |  | | |
| May | 0.042 | 0.329 | 0.129 | 0.897 |  | |  |  | | |
| June | 0.246 | 0.317 | 0.776 | 0. 348 |  | |  |  | | |
| July | 0.246 | 0.320 | 0.636 | 0.525 |  | |  |  | | |
| August | 0.670 | 0.298 | 2.251 | 0.024 |  | |  |  | | |
| September | 0.858 | 0.291 | 2.945 | 0.003 |  | |  |  | | |
| October | 0.494 | 0.305 | 1.621 | 0.105 |  | |  |  | | |
| Position (ground) | 0.747 | 0.123 | 6.060 | < 0.001 |  | |  |  | | |

Table S3. Output of the best generalized linear mixed model explaining invertebrate predation rates on *Vachellia* trees in Evrona Nature Reserve, southern Israel, during 2018-2019.

|  | **Effect** | | | | | | |  |  | |
| --- | --- | --- | --- | --- | --- | --- | --- | --- | --- | --- |
|  | **Fixed:** | | | |  | **Random  (tree ID, n = 30):** | | | |  |
|  | **Estimate** | **SE** | **z-value** | **p-value** |  | | **Variance** | **St. Dev** | | |
| (Intercept) | -3.835 | 0.375 | -10.22 | < 0.001 |  | | 0.034 | 0.185 | | |
| January | 1.311 | 0.415 | 3.158 | 0.002 |  | |  |  | | |
| February | 1.619 | 0.405 | 3.996 | < 0.001 |  | |  |  | | |
| March | 1.526 | 0.409 | 3.734 | < 0.001 |  | |  |  | | |
| April | -0.003 | 0.51 | -0.006 | 0.995 |  | |  |  | | |
| May | 0.656 | 0.449 | 1.46 | 0.144 |  | |  |  | | |
| June | 0.86 | 0.437 | 1.969 | 0.049 |  | |  |  | | |
| July | 1.034 | 0.428 | 2.417 | 0.016 |  | |  |  | | |
| August | 1.269 | 0.417 | 3.043 | 0.002 |  | |  |  | | |
| September | 1.393 | 0.412 | 3.379 | 0.001 |  | |  |  | | |
| October | 1.398 | 0.412 | 3.39 | 0.001 |  | |  |  | | |
| Position (ground) | 0.791 | 0.142 | 5.556 | < 0.001 |  | |  |  | | |

| Table S4. Output of the post-hoc test with Holm correction for the factor “month” in the generalized linear mixed model for invertebrate predation rate. The test was performed via the R package *lsmeans*. Significant comparisons are given in bold. |
| --- |

| **Contrast** | **Estimate** | **SE** | **z-value** | **Significance** |
| --- | --- | --- | --- | --- |
| November - January | -1.3115 | 0.415 | -3.158 | 0.073 |
| **November - February** | -1.6192 | 0.405 | -3.996 | 0.0035 |
| **November - March** | -1.5255 | 0.409 | -3.734 | 0.0098 |
| November - April | 0.00325 | 0.51 | 0.006 | 1 |
| November - May | -0.6557 | 0.449 | -1.46 | 1 |
| November - June | -0.8599 | 0.437 | -1.969 | 1 |
| November - July | -1.0336 | 0.428 | -2.417 | 0.6128 |
| November - August | -1.2686 | 0.417 | -3.043 | 0.1032 |
| **November - September** | -1.393 | 0.412 | -3.379 | 0.0349 |
| **November - October** | -1.3975 | 0.412 | -3.39 | 0.0349 |
| January - February | -0.3078 | 0.277 | -1.111 | 1 |
| January - March | -0.2141 | 0.282 | -0.76 | 1 |
| January - April | 1.31471 | 0.415 | 3.167 | 0.0725 |
| January - May | 0.65576 | 0.338 | 1.94 | 1 |
| January - June | 0.45155 | 0.321 | 1.405 | 1 |
| January - July | 0.27792 | 0.309 | 0.9 | 1 |
| January - August | 0.04285 | 0.294 | 0.146 | 1 |
| January - September | -0.0816 | 0.287 | -0.284 | 1 |
| January - October | -0.086 | 0.287 | -0.3 | 1 |
| February - March | 0.0937 | 0.267 | 0.351 | 1 |
| **February - April** | 1.62249 | 0.405 | 4.004 | 0.0034 |
| February - May | 0.96354 | 0.326 | 2.959 | 0.1329 |
| February - June | 0.75933 | 0.308 | 2.462 | 0.5658 |
| February - July | 0.58569 | 0.295 | 1.983 | 1 |
| February - August | 0.35063 | 0.28 | 1.254 | 1 |
| February - September | 0.22622 | 0.272 | 0.83 | 1 |
| February - October | 0.22174 | 0.272 | 0.814 | 1 |
| **March - April** | 1.52879 | 0.408 | 3.743 | 0.0096 |
| March - May | 0.86984 | 0.33 | 2.638 | 0.3501 |
| March - June | 0.66563 | 0.313 | 2.129 | 1 |
| March - July | 0.49199 | 0.3 | 1.641 | 1 |
| March - August | 0.25692 | 0.284 | 0.904 | 1 |
| March - September | 0.13252 | 0.277 | 0.478 | 1 |
| March - October | 0.12804 | 0.277 | 0.462 | 1 |
| April - May | -0.659 | 0.449 | -1.467 | 1 |
| April - June | -0.8632 | 0.437 | -1.977 | 1 |
| April - July | -1.0368 | 0.428 | -2.425 | 0.6128 |
| April - August | -1.2719 | 0.417 | -3.051 | 0.1026 |
| **April - September** | -1.3963 | 0.412 | -3.388 | 0.0349 |
| **April - October** | -1.4008 | 0.412 | -3.398 | 0.0346 |
| May - June | -0.2042 | 0.364 | -0.561 | 1 |
| May - July | -0.3779 | 0.353 | -1.07 | 1 |
| May - August | -0.6129 | 0.34 | -1.802 | 1 |
| May - September | -0.7373 | 0.334 | -2.206 | 1 |
| May - October | -0.7418 | 0.334 | -2.219 | 1 |
| June - July | -0.1736 | 0.337 | -0.515 | 1 |
| June - August | -0.4087 | 0.324 | -1.263 | 1 |
| June - September | -0.5331 | 0.317 | -1.679 | 1 |
| June - October | -0.5376 | 0.318 | -1.693 | 1 |
| July - August | -0.2351 | 0.311 | -0.755 | 1 |
| July - September | -0.3595 | 0.305 | -1.179 | 1 |
| July - October | -0.364 | 0.305 | -1.194 | 1 |
| August - September | -0.1244 | 0.29 | -0.43 | 1 |
| August - October | -0.1289 | 0.29 | -0.445 | 1 |
| September - October | -0.0045 | 0.283 | -0.016 | 1 |

| Table S5. Output of the best generalized linear mixed model explaining vertebrate predation rates on *Vachellia* trees in Evrona Nature Reserve, southern Israel, during 2018-2019. |
| --- |

| **Effects** |  | | | | | | | | |
| --- | --- | --- | --- | --- | --- | --- | --- | --- | --- |
| **Fixed:** | | | | | |  | **Random**  **(tree ID, n = 30):** | |  |
|  | | **Estimate** | **SE** | **z-value** | **p-value** |  | **Variance** | **St. Dev** | |
| (Intercept) | | -3.956 | 0.247 | -15.993 | < 0.001 |  | 0.129 | 0.359 | |
| Treatment (oil) | | 0.541 | 0.261 | 2.074 | 0.0381 |  |  |  | |
| Position (ground) | | 0.433 | 0.221 | 1.961 | 0.0499 |  |  |  | |
